# Microbial survival strategies in desiccated roots of *Myrothamnus flabellifolia*

**DOI:** 10.3389/fmicb.2025.1560114

**Published:** 2025-03-28

**Authors:** Shandry M. Tebele, Rose A. Marks, Jill M. Farrant

**Affiliations:** ^1^Department of Molecular and Cell Biology, University of Cape Town, Rondebosch, South Africa; ^2^Department of Plant Biology, University of Illinois Urbana-Champaign, Urbana, IL, United States

**Keywords:** desiccation, metatranscriptomics, microbiomes, *Myrothamnus flabellifolia*, plant growth-promoting bacteria, resurrection plants, rhizosphere

## Abstract

**Introduction:**

Root-associated microbiomes are critical to plant vigor, particularly under drought stress. The spatial dynamics of microbial community diversity and composition are strongly influenced by plant root and environmental factors. While the desiccation tolerance of the resurrection plant *Myrothamnus flabellifolia* using leaf tissue has been previously investigated, the transcriptional responses of its root-associated microbiomes under desiccation remain completely unexplored.

**Methods:**

Here, we conducted metatranscriptome sequencing on root samples of *M. flabellifolia* collected in the field across four states: dry, desiccated, partially hydrated, and fully hydrated.

**Results:**

Bacterial transcripts dominated the root metatranscriptome across all conditions. Desiccated roots exhibited a significant increase in transcripts from Actinomycetota, whereas fully hydrated roots showed an enrichment of Pseudomonadota. Under desiccation, root-associated bacteria upregulated genes involved in antioxidant systems, trehalose biosynthesis, and hormonal regulation.

**Discussion:**

These findings highlight microbial adaptive mechanisms to withstand extreme water loss. In contrast, the bacterial transcriptional response in hydrated roots was characterized by genes linked to peptidoglycan biosynthesis, sugar transporters, and chemotaxis. Taken together, our findings indicate that root-associated bacteria deploy defense mechanisms analogous to those of their host plant to adapt to extreme drought stress, highlighting their crucial role in plant resilience.

## 1 Introduction

Plants and microbiota have evolved adaptive mechanisms to combat the negative impacts of drought. Recent studies highlight the critical interplay of plant microbe interactions in improving performance under drought conditions (Gholizadeh et al., 2024; Shaffique et al., 2022). For instance, microbes associated with roots of plants from semi-arid environments prone to extreme drought, function synergistically with plants to enhance drought tolerance (Du et al., 2024). A deeper understanding of the complex interactions between plant roots, microorganisms, and the adaptive mechanisms they employ is crucial for understanding survival in the face of drought.

Under drought stress, specific microbes dominate the soil microbiome. Specifically, monoderm bacteria, which are primarily gram-positive, increase under drought, possibly due to their thick peptidoglycan cell walls, while diderm lineages (gram-negative) decrease during drought (Xu et al., 2018). Changes in the community composition also impact the function of the soil microbiome under drought. A recent metatranscriptome study demonstrated that moderate drought activated oxidative, osmotic and heat stress-related genes in rhizosphere bacteria such as *Burkholderiales* and *Rhodospirillales* (Tartaglia et al., 2023). Presumably, these stress responsive genes combat osmotic stress in the microbes, however, they might also enhance the drought tolerance of host plants through synergistic effects. For instance, Actinomycetota have been shown to improve drought tolerance in plants by modulating secondary metabolite biosynthesis, carbohydrate metabolism, and amino acid transportation (Dong et al., 2025; Xu et al., 2018). In these and other examples, interaction with specific root-associated microbes positively impacts plant growth and resilience.

Most studies focus on moderate drought (i.e., dehydration but not desiccation) (Gao et al., 2024; Grzyb and Skłodowska, 2022) and the effects of extreme water limitation on plant microbe interactions are not well characterized. *Myrothamnus flabellifolia* is a well-known “resurrection plant” and a valuable model for studying extreme water limitation due to its ability to survive for prolonged periods of up to a year in the desiccated stated (Ginbot and Farrant, 2011; Farrant and Kruger, 2001). *Myrothamnus flabellifolia* has captivated the attention of traditional healers and scientists for decades, and much is known about the mechanisms of desiccation tolerance in its leaves (Marks et al., 2022). For instance, desiccation responses of *M. flabellifolia* include leaf folding and the production of polyphenols, such as anthocyanins, which limit excess light and reduce photooxidation in the chloroplast (Bentley and Farrant, 2020; Kranner et al., 2002; Moore et al., 2006, 2007). In parallel, the accumulation of antioxidants helps mitigate oxidative stress by scavenging reactive oxygen species (ROS), while non-reducing sugars stabilize cellular structures by replacing water molecules through hydrogen bonding, thereby preserving the conformation of macromolecules (Farrant, 2007; Bentley et al., 2019). However, little is known about the root-associated microbiomes of this and other resurrection plants and their mechanisms of tolerance (Tebele et al., 2021).

Recently, researchers have begun to study the root microbiome of resurrection plants, due to their potential to facilitate desiccation tolerance of the parent plant. To date, the microbiomes of only four resurrection plants have been characterized including *Boea hygrometrica* (Sun et al., 2024), *M. flabellifolia* (Tebele et al., 2024), *Ramonda* spp. (Djokic et al., 2010; Lozo et al., 2023; Rakić et al., 2013), and *Tripogonella spicata* (Silva et al., 2023a,b). These studies demonstrated that resurrection plants host a diversity of beneficial microbial communities with complex defense mechanisms that may impact the host plants.

The functional dynamics of how root-associated microbiomes contribute to the overall tolerance of the plant holobiont remain complex and poorly understood. Previous studies on the resurrection plant microbiome have primarily focused on describing microbial diversity and composition. In this study, however, we delve deeper into the desiccation mechanisms employed by these microbes at the transcriptional level and explore how these mechanisms are similar to or differ from those of the host plant. Here, we hypothesized that the soil microbiome of *M. flabellifolia* may employ a similar molecular response to desiccation as its host. To test this, we characterized transcriptional changes in the root-associated microbiome of *M. flabellifolia* under desiccation stress and upon rehydration.

## 2 Materials and methods

### 2.1 Sampling procedure

**Figure 1:**
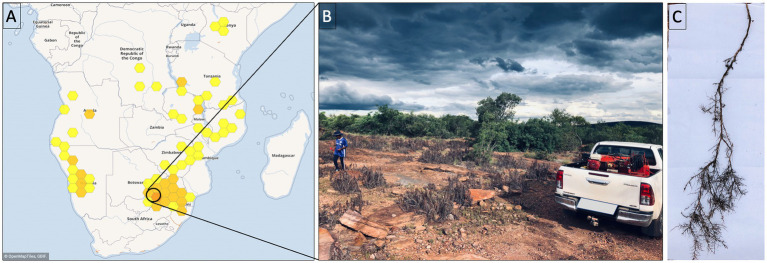
**(A)** Geographical distribution of *Myrothamnus flabellifolia*. **(B)** Natural habitat of *M. flabellifolia* at the SwebeSwebe field site. **(C)** Root anatomy of *M. flabellifolia* showing root hairs under desiccated conditions.

*Myrothamnus flabellifolia* is a woody shrub in the eudicot order Gunnerales, growing up to 1.5 m in height and is often found on rock inselbergs as shown in Figures 1A,B (Glen et al., 1999; Moore et al., 2007). In the current study, the roots of *M. flabellifolia* were sampled at SwebeSwebe Private Nature Reserve in Limpopo, South Africa, during a natural dehydration event. The relative water content (RWC) of leaves and roots was measured as described in Barrs and Weatherley (1962). Root samples were collected at four-time points: partially dry (PD) with a RWC of 39.78 ± 6.55%, desiccated (D) with a RWC of 10.86 ± 0.64%, partially rehydrated (PR) with a RWC of 43 ± 7.72% and fully rehydrated (FR) with a RWC of 81.25 ± 3.77%. The values represent the mean percentage and standard error, respectively. Six biological plant replicates (3 males and 3 females) were sampled at each time point. These root tissues were shaken vigorously to remove bulk and the rhizosphere soil. RNA molecules are unstable and rapidly degrade, therefore, no further sample washing was applied. Samples were immediately flash-frozen in liquid nitrogen. These roots with small particles of soil were used for metatranscriptomic analysis of root-associated bacteria (Figure 1C). Therefore, the identified microbial communities emanate from the endosphere (within root) and rhizoplane (root surface).

### 2.2 RNA isolation and metatranscriptomic sequencing

The frozen roots from each sampling point were ground in liquid nitrogen using a sterile pestle and mortar, followed by a Retsch mixer mill MM 400 to break down rigid cell walls via rigorous oscillation for 10 min at a frequency of 28 Hz. Total RNA from roots was isolated according to Majidi and Bahmani (2017) with minor modifications. Briefly, a 3% Cetyltrimethylammonium bromide (CTAB) extraction buffer was used together with beta-mercaptoethanol. The RNA quantity was further analyzed using a Qubit 4.0 Fluorometer with fluorescent-based Qubit RNA Broad Range Assay Kit (Thermo Fisher Scientific). Out of 24 root samples only 18 passed the quality parameters and they were sent for metatranscriptomic sequencing at the Centre for Proteomic and Genomic Research (CPGR). The RNA integrity was assessed using Agilent TapeStation 4,200. Libraries for 18 samples were prepared using the Illumina Stranded Total RNA Prep kit and were ribodepleted by Ribo-Zero Plus, which removes the redundant ribosomal RNA of prokaryotic and eukaryotic cells according to manufacturer’s protocol. Libraries were sequenced on a NovaSeq 6,000 platform with a paired-end run of 2 × 100 bp by CPGR.

### 2.3 Bioinformatic and statistical analysis

Metatranscriptome sequencing of 18 root samples generated 981.64 gigabytes (GB) of sequence data. The samples were preprocessed through a metatranscriptomic pipeline (samsa) with minor alterations (Westreich et al., 2018). The BBTool package’s bbduk.sh component^1^ was employed for k-mer matching, quality trimming, removal of adaptor sequences and low-quality reads below a 35 phred score. FastQC assessed the quality of the trimmed reads, and quality reporting for all samples was generated from MultiQC.^2^ The ribodetector tool (Deng et al., 2022) was used to eliminate ribosomal RNA (rRNA) sequence residues not depleted by the Ribo-Zero Plus kit (Illumina, United States). Reads that passed QC were co-assembled into transcripts and annotated using OmicsBox.^3^ Samples were co-assembled using megahit with iterative kmers size 31, 41, 51, 61, 71, 81, 91 bases (Pande et al., 2023). Transcript prediction and annotation was performed by calling transcripts for each assembled contig using Prodigal v2.6.2 and transcripts were then assigned KEGG orthologs (Kanehisa and Goto, 2000). EggNOG database (Huerta-Cepas et al., 2019) was used to annotate transcripts as either bacteria, fungi, plants or viruses at various taxa levels. Additionally, SEED subsystems were employed to identify upregulated biological processes or pathways through the analysis of the predicted prokaryotic protein sequences. The SEED subsystems are developed as a tool to assign functional roles to the microbiome, utilizing an integrated database from various sources and is predominantly employed in metatranscriptomics and shotgun sequencing studies (OverBeek et al., 2014).

All downstream statistical analysis was performed using R software v4.2.0. Principal component analysis (PCA) of bacterial taxonomic composition and total gene expression were used to determine the overall consistency across replicates and variation of the expressed transcripts between dehydrated and rehydrated samples. Taxonomic data were normalized by total sum scaling. DESeq2 R package version 1.36 was used to identify differentially abundant transcripts (DATs) and taxonomic abundances through Wald test (*p* < 0.05) and negative binomial linear models with a log2 fold change cut-off of 2 between dehydrated and rehydrated conditions were employed (Supplementary Table S1). Subsequently, volcano and heatmap plots of DATs were generated using adjusted *p* < 0.05. A one-way analysis of variance (ANOVA) test was used to determine the statistical difference (*p* < 0.01) of RWC between dehydrated and rehydrated plant tissues.

## 3 Results and discussion

### 3.1 Multivariate analysis of the global metatranscriptome

**Figure 2:**
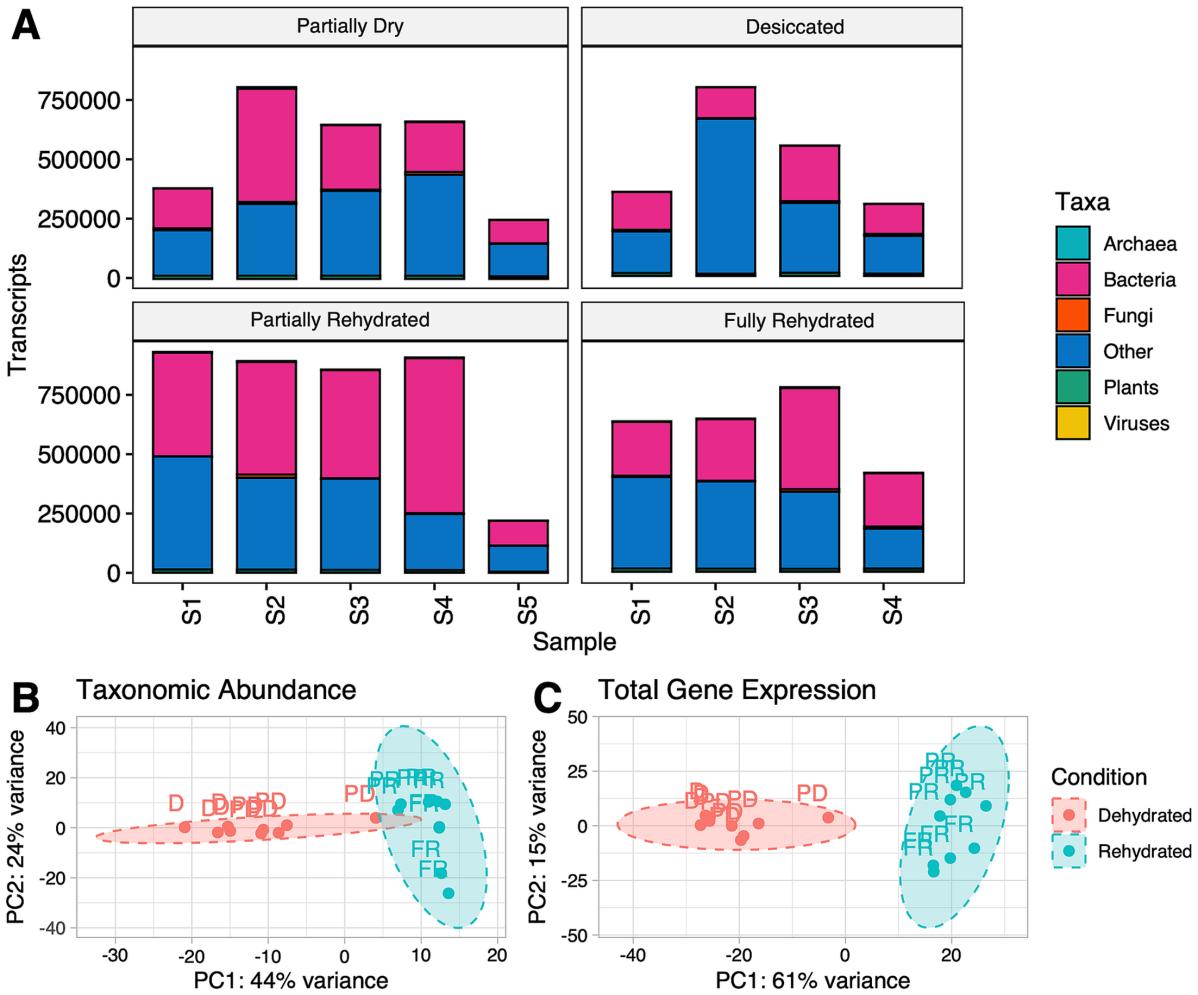
**(A)** Kingdom-level taxonomic affiliation of the transcripts retrieved from all samples (S1-S5) across different conditions excluding unknown transcripts. **(B)** Principal component analysis (PCA) of bacterial transcripts showing taxonomic abundance and **(C)** total gene expression. Dehydrated conditions include partially dehydrated (PD) and desiccated (D). Rehydrated samples were labeled as partially rehydrated (PR) and fully rehydrated (FR).

To better understand desiccation tolerance, it is essential to investigate not only the aerial tissues of a plant, but also the roots to gain insights into the interactions between roots and soil microenvironment. The metatranscriptome of 18 roots samples across four-time points generated a total of 147,996,445 clean and assembled reads (overall mean: 8,222,024, max: 13,116,483, min 867,317). Among the transcripts, 46.5% were assigned to bacteria, 1.8% to plants, 1.0% to fungi, 0.4% to Archaea, 0.2% to viruses, and 50.1% were unclassified and are represented as others (Figure 2A). Almost half of the transcripts belonged to bacterial taxa and the other half was unclassified. These results are similar to the study by Pande et al. (2023), also reported high levels bacterial transcripts in wheat roots under drought stress. We should not discount the small percentage of transcripts derived from fungi, archaea, viruses, and plants that also contribute to the overall dataset. However, this study focused primarily on the bacterial transcripts due to their prominence in the dataset.

A PCA was used to assess broad patterns of taxonomic and transcript abundance across dehydrated and rehydrated samples. Dehydrated (partially and fully desiccated) and rehydrated (partially and fully) samples formed two distinct clusters in PCA plots (Figure 2B). Consequently, downstream analyses focused on the primary comparison between the two broad categories of dehydration and rehydration. PCA explained a large proportion of the variability in the dataset and separated samples clearly by hydration status for both taxonomic and transcript abundances. The first principal component accounted for 44% of the variability, and the second accounted for 24% of the variability in the taxonomic dataset (Figure 2B). Similar trends were observed for transcript abundance, with PC1 accounting for 61% of the variability and PC2 accounting for another 15% with clear separation between dehydrating and rehydrating samples (Figure 2C). These findings suggest that distinct microbial taxa and transcripts dominate during dehydration compared to rehydration.

### 3.2 Influence of low RWC on taxonomic abundance

**Figure 3:**
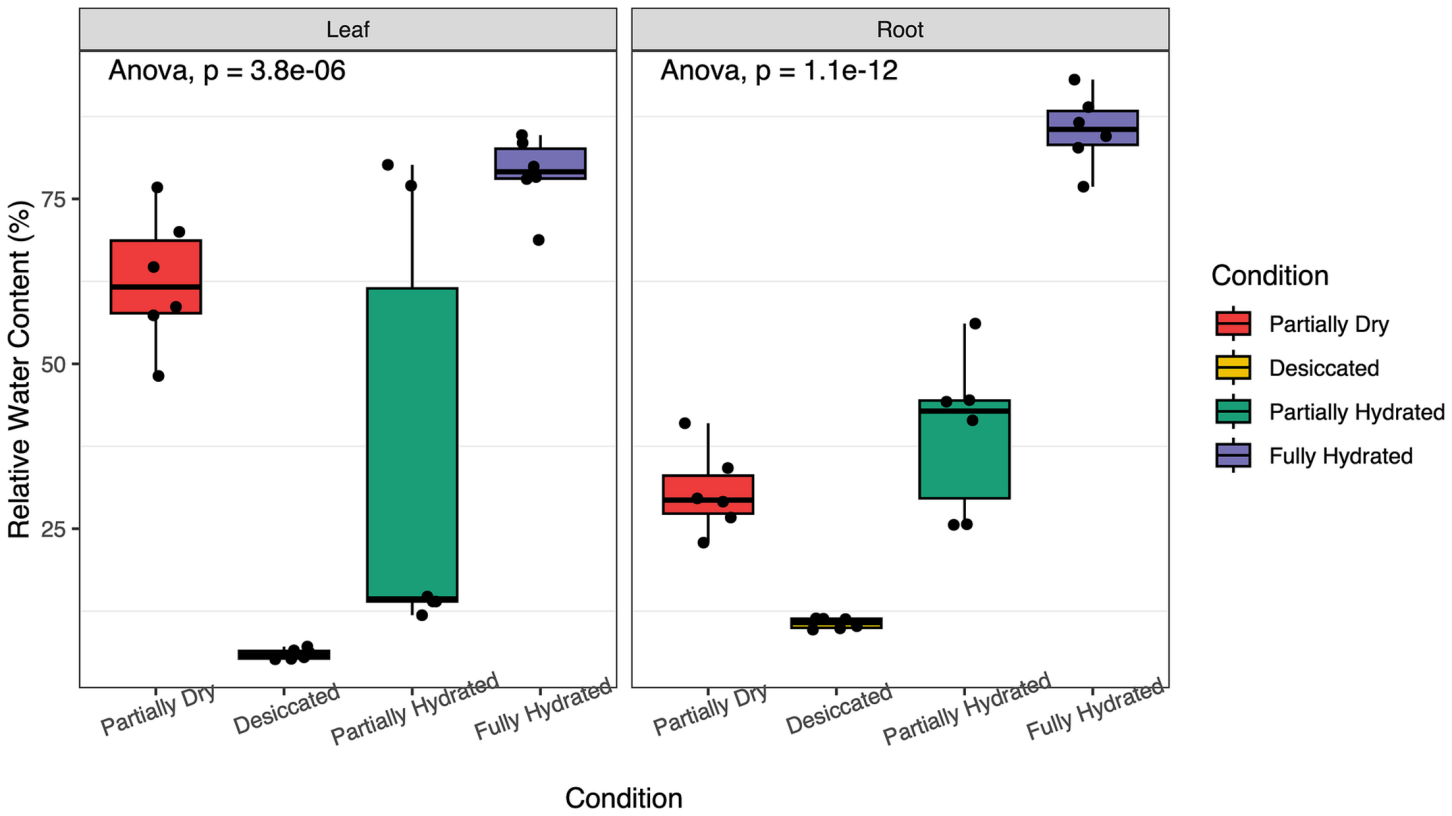
Effects of dehydration and rehydration on the relative water content (RWC) of leaf and root tissues of *Myrothamnus flabellifolia* in Swebeswebe field site. Plants were sampled at four-time points during the process of dehydration and rehydration.

This study explored how the root microbiome of *M. flabellifolia* responds to fluctuations in water availability. In field conditions, drought stress significantly reduced the RWC of leaves and roots (Figure 3). Across four sampling time points, RWC varied significantly (*p* < 0.001), decreasing during desiccation and recovering upon rehydration (Figure 3). Although this study primarily focuses on the root dynamics, leaf RWC was measured to corroborate the rate of water loss in the shoot tissue and link the current study with previous work on the leaf tissue of *M. flabellifolia*.

**Figure 4:**
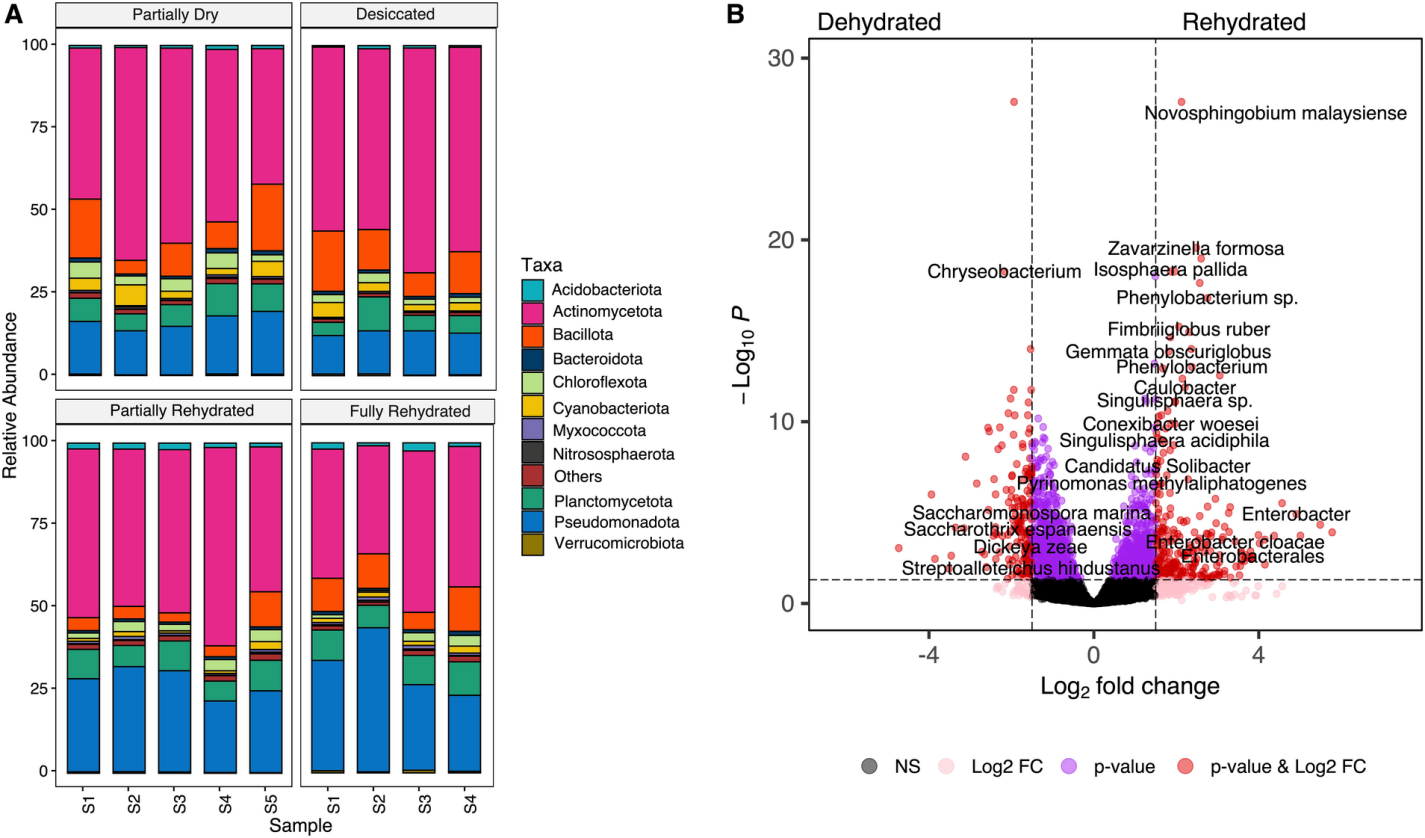
**(A)** Bacterial taxonomic profiling of transcripts at phylum level across four-time points. **(B)** Differentially abundant transcripts of associated bacterial species under dehydrated (partially dry and desiccated) and rehydrated (partially and fully rehydrated) conditions.

The reduction in root RWC was associated with shifts in the microbial community during dehydration and rehydration. Microbial taxa were classified at the phylum and species level based on the sequenced transcripts. The relative abundances of Actinomycetota, Bacillota, Cyanobacteriota, and Chloroflexota were significantly higher in PD (39.78 ± 6.55 RWC) and D samples (10.86 ± 0.64 RWC), but rapidly declined upon rehydration. In contrast, a high RWC (81.25 ± 3.77) of FR samples led to a significant increase in Pseudomonadota compared to dehydrated roots (Figure 4A). A substantial number of transcripts identified in this study were derived from monoderm lineages, particularly Actinomycetota and Bacillota, suggesting their potential ability to survive desiccation. These results align with previous findings that these bacterial lineages increase in relative abundance in both the rhizosphere soil and endosphere of resurrection plants during desiccation (Sun et al., 2024; Tebele et al., 2024). *Actinomycetes* species are known to produce antimicrobial compounds (Dávila Costa and Amoroso, 2014) and may also enhance drought tolerance in host plants by expressing hydrolase genes (Wang et al., 2019). Recent research indicates that beneficial root-associated microbes not only promote plant growth but also play a key role in regulating responses to environmental stress (de Albuquerque et al., 2022; Qin et al., 2015; Salimi et al., 2023; Tao et al., 2007; Timmusk and Wagner, 1999). *M. flabellifolia* may have evolved the ability to recruit and sustain microbes with co-adapted drought resilience mechanisms. Consistent with previous studies on resurrection plant microbiomes (Lozo et al., 2023; Sun et al., 2024), our findings highlight significant shifts in dominant root-associated microbes under drought stress. In contrast, drought stress had a minor impact on fungal microbiomes, likely due to high variability in their relative abundance across replicates. Ascomycota, Basidiomycota, Glomeromycota, and Mucoromycota were consistently present across all timepoints (Supplementary Figure S1).

This study identified a total of 1764 species with significant changes in abundance across the time-course. Of these, 859 species (9.5%) were significantly enriched (*p* < 0.05) in dehydrated roots (10.86 ± 0.64 RWC), while 906 species (10%) increased in abundance in rehydrated samples (81.25 ± 3.77 RWC) (Figure 4B). At the species level, the most significantly increased transcripts under dehydrated conditions belonged to *Azospirillum halopraeferens*, *Amycolatopsis benzoatilytica*, *Chryseobacterium*, *Eggerthellaceae* spp., *Ehrlichia ruminantium*, *Kibdelosporagium* spp., *Myroides odoratus*, *Nostoc* spp., *Rhizobium anhuiense*, *Rickettsiales* spp., *Saccharomonospora marina*, *Tolypothrix* spp., and *Streptoalloteichus hindustanus* (Figure 4B). Many of these species are classified as plant growth-promoting bacteria (PGPB) known to enhance drought tolerance in host plants (Glick, 2012; Tartaglia et al., 2023). Notably, Cyanobacteriota phylum, was enriched under dehydration, including species such as *Leptolyngbya* and *Nostoc* spp., which were also detected in desiccated *M. flabellifolia* roots (Tebele et al., 2024). These findings align with Liu et al. (2021), who observed Cyanobacteriota enrichment in rhizosheath soil of switchgrass under drought stress. Cyanobacteria possess key drought-adaptive traits, including water retention via exopolysaccharide secretion, soil stabilization, and participation in carbon and nitrogen cycling (Adams and Duggan, 2008; Lebre et al., 2017; GR et al., 2021). These attributes position them as crucial contributors to drought mitigation and sustainable agriculture.

### 3.3 Oxidative and osmotic stress response

**Figure 5:**
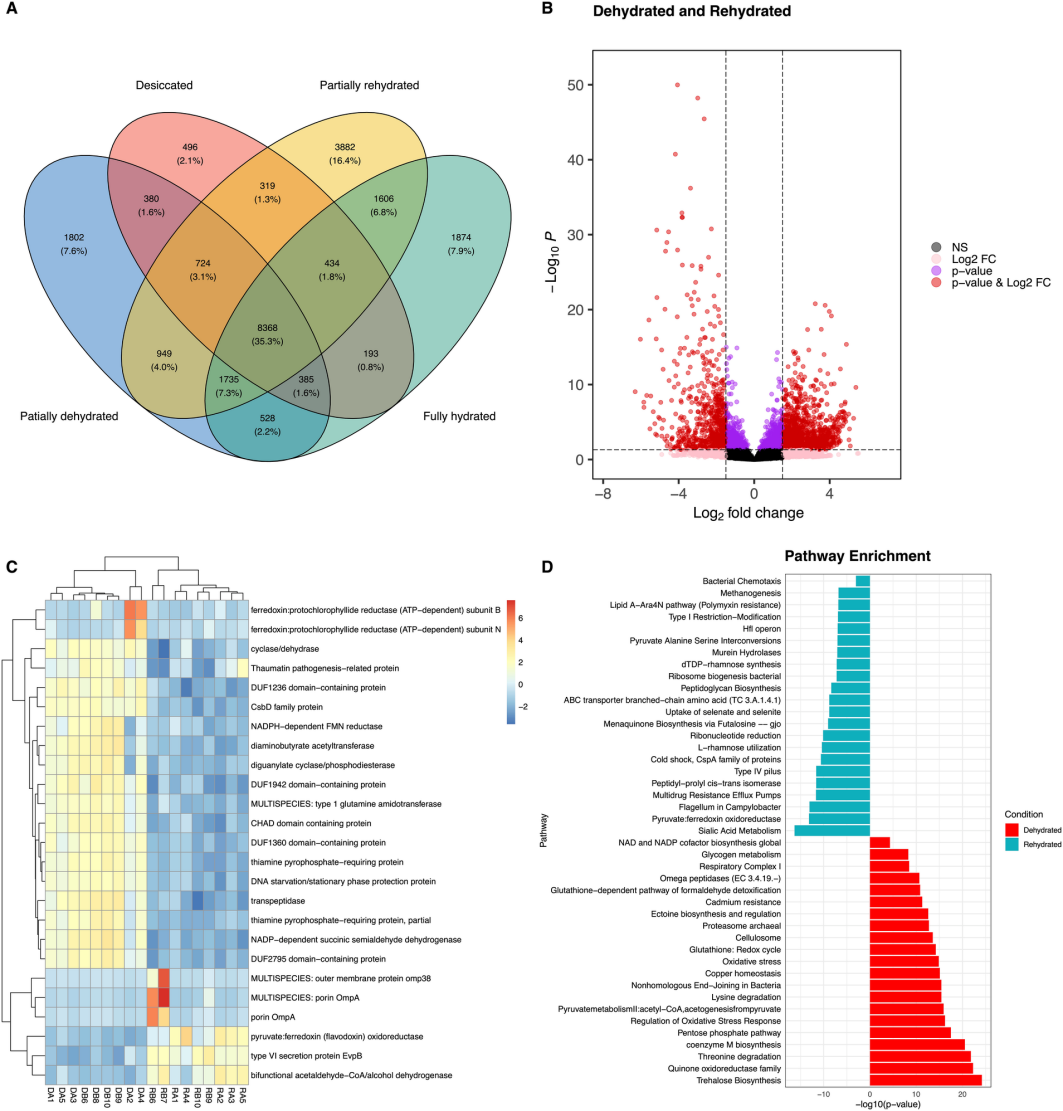
Venn diagram shows the unique and shared transcripts across four conditions **(A)**. Differentially abundant transcripts (DATs), significantly transcripts are highlighted in red with an application of adjusted *p*-value <0.05 and log fold change cut-off = 2 **(B)**. Heatmap of the top 25 DATs of bacteria in roots of *Myrothamnus flabellifolia,* DA1-DA4 represent dehydrated and RB6-RA5 is rehydrated condition **(C)**. Functional assignment of bacterial transcripts associated with roots under dehydration (red) and rehydration (cyan) conditions **(D)**.

**Figure 6:**
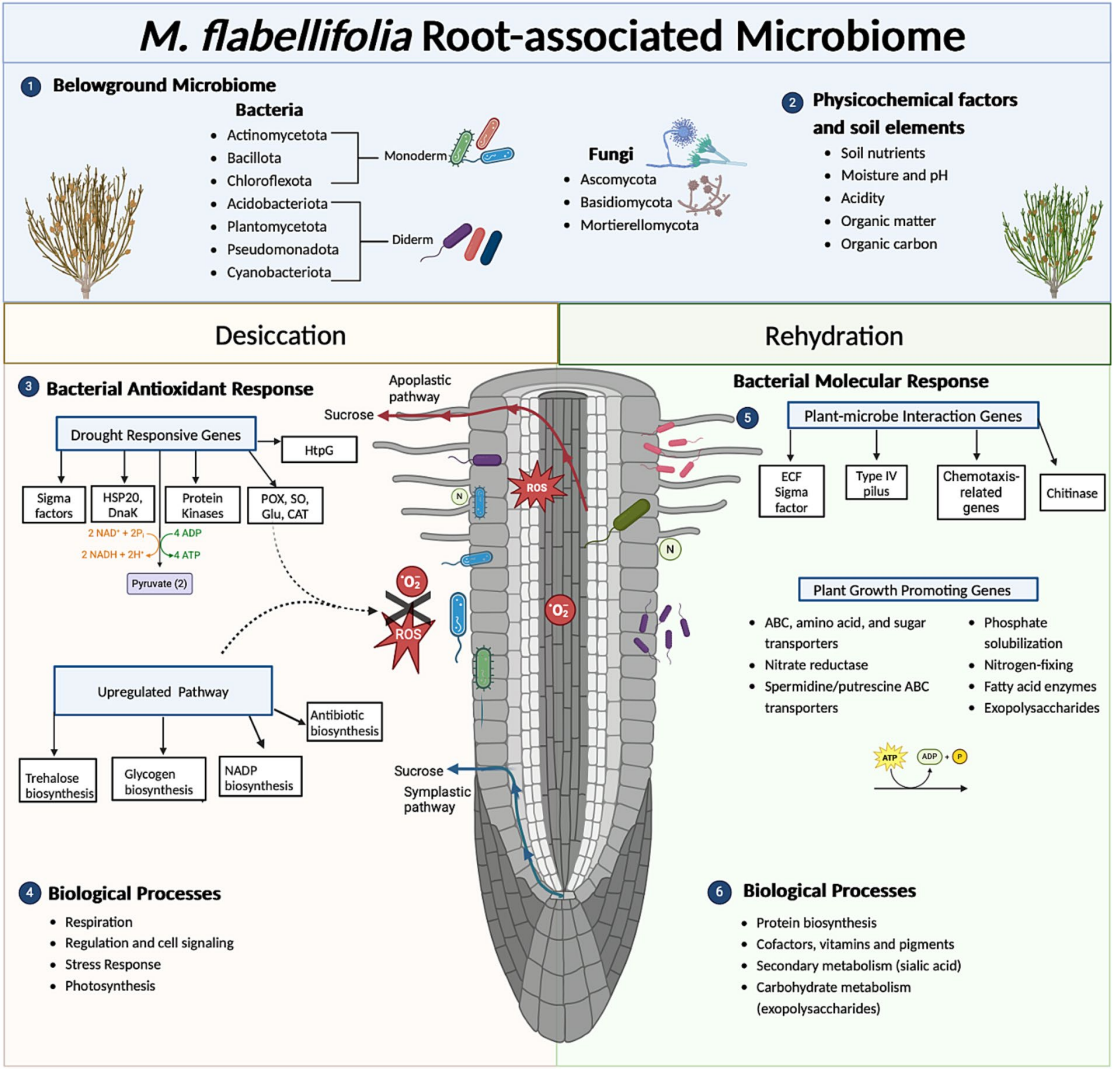
Summary diagram of key findings of functional role(s) of the root-associated microbiome, bacteria in particular, of *Myrothamnus flabellifolia*. The abundance of sucrose in *M. flabellifolia* roots under drought stress may be secreted into the rhizosphere via apoplastic or symplastic pathways, potentially acting as a chemoattractant for the microbial community (Tebele, 2024). (1) Metatranscriptomic analysis identified predominant bacterial communities in *M. flabellifolia* roots under desiccated conditions (<10% RWC). This is consistent with findings from Tebele et al. (2024), which utilized amplicon-based metagenomics to quantify similar microbiomes in bulk soil, rhizosphere, and endosphere compartments. (2) The rhizosphere soil of *M. flabellifolia* exhibited enhanced physicochemical properties and nutrient availability compared to bulk soil, potentially attributed to the activity of the microbiome during desiccation stress (Tebele et al., 2024). (3) Differentially abundant transcripts (DATs) in bacterial cells under desiccation stress were associated with drought response mechanisms. Key pathways included the activation of sigma factors for extracellular stress detection, upregulation of drought-responsive genes (e.g., molecular chaperones such as DnaK and HSP20), ATP synthase, antioxidant enzymes, protein kinases, and carbohydrate biosynthesis to mitigate reactive oxygen species (ROS). (4) Biological processes enriched within root-associated bacterial cells under desiccation included respiration, stress responses, and photosynthesis. Notably, photosynthetic bacteria, such as Cyanobacteria spp., were implicated in these functions. (5) Upon rehydration, bacterial molecular responses showed upregulation of DATs associated with plant-microbe interactions, including genes involved in chemotaxis and exopolysaccharide biosynthesis. (6) Favorable conditions also enhanced the abundance of plant growth-promoting genes, including those related to nutrient recycling, sugar and amino acid transport, and the regulation of metabolite flux in bacterial cells. DATs under rehydration were further linked to biological processes such as protein biosynthesis (e.g., amino acid transporters) and carbohydrate metabolism (e.g., exopolysaccharide synthesis, trehalose utilization), underscoring the role of the microbiome in promoting resilience and recovery of *M. flabellifolia* after desiccation stress.

Oxidative stress intensifies with prolonged desiccation, becoming increasingly detrimental to both plants and microbes. Under dehydration conditions, the most abundant DATs were linked to respiratory metabolism, accounting for 24.32% of the total transcripts. These included genes encoding respiratory-chain NADH dehydrogenase subunit 1, pyruvate oxidase, NAD(+)/NADH kinase, apoprotein A2, and cytochrome oxidase proteins (Figure 5C; Supplementary Figure S2). Notably, a cluster of orthologous groups (COG) analysis revealed that many DATs in dehydrated roots were associated with Actinomycetota (Supplementary Table S2). The upregulated genes under drought also included a wide spectrum of ROS scavenging systems such as NAD-glutamate dehydrogenase, superoxide dismutase and manganese catalase and peroxidase (Figure 6), which play essential roles in detoxification and cellular homeostasis (Wang et al., 2020). NADKs function as key components of antioxidant systems and are the only enzymes that catalyze the phosphorylation of NADH to generate NADPH in almost all living organisms (Li et al., 2018), which potentially enhance the desiccation tolerance of bacteria. Additionally, the expression of indole-pyruvate dehydrogenase genes suggests bacterial synthesis of indole-3-acetic acid (IAA), which may promote root growth in *M. flabellifolia* (Figure 1D). This is consistent with previous studies reporting drought responsive gene expression in PGPB, such as *Azospirillum* spp., *Bacillus*, and *Rhizobium* spp. (Akbar et al., 1999; Kim et al., 2019; Xu et al., 2020; Zhang et al., 2021; Zhu et al., 2022). Although bacterial gene expression is primarily an adaptation to counteract drought stress for the bacteria themselves, these defense mechanisms may also enhance the fitness and survival of the host plant under harsh environmental conditions.

As expected, stress response pathways were more abundant in dehydrated samples (10.86 ± 0.64 RWC) than in rehydrated ones (81.25 ± 3.77 RWC) (Figure 5D), with the upregulation of molecular chaperone genes such as DnaK and HtpG, along with HSP20, proline/glycine betaine, glutamate decarboxylase, superoxide dismutase, catalase, peroxidase, and NAD-glutamate dehydrogenase genes (Figure 5C). The expression of DnaK genes has also been reported in *Nostoc* spp. during dehydration, where they play a role in protecting photosystem II membranes (Xu et al., 2020). Our findings suggest that these molecular chaperones play a key role in stabilizing cell membranes against dehydration-induced damage, with their expression regulated by intracellular water status. Interestingly, resurrection plants such as *M. flabellifolia* also exhibit high expression of antioxidant enzymes during desiccation (Kranner et al., 2002), indicating that protective mechanisms against oxidative stress may be conserved from bacteria to vascular plants. This study detected a broad range of ROS-scavenging systems, including antioxidant enzymes and molecular chaperones, which are critical for sustaining cellular homeostasis within bacterial cells.

Water deficit conditions disrupt biological and metabolic processes of both microbes and plants, leading to osmotic stress, loss of cell membrane integrity, and an increase in ROS (Farrant et al., 2009; Grzyb and Skłodowska, 2022; Malik and Bouskill, 2022). The latter could trigger the upregulation of bacterial genes involved in cell signaling and responses to extracellular environmental stimuli (Supplementary Figure S2). These findings align with a previous study on the transcriptome of *Burkholderia phytofirmans*, which demonstrated that drought stress activated signal transduction systems—typically consisting of histidine kinase membrane sensors (Sheibani-Tezerji et al., 2015). Signal transduction systems are essential for detecting water-limiting conditions and initiating regulatory cascades that drive gene expression related to environmental stress responses (Kocharunchitt et al., 2012). In this study, protein kinases such as DUF-related and leucine-rich repeat kinases were found to be abundant in drought stress (Figure 5C). Notably, these receptor-like kinases were also upregulated in the leaves of *M. flabellifolia* under drought conditions (Ma et al., 2015) and are known to transmit osmotic signals in plants (Chen et al., 2021). This finding suggests that root-associated bacteria may utilize mechanisms similar to those of their host plants, including the upregulation of glutathione metabolism and the pentose phosphate pathway, to withstand cycles of drying and rehydration.

### 3.4 Functions of differentially abundant transcripts and biological metabolisms

Among the 28,290 annotated transcripts, PD samples with an RWC of 39.8% exhibited a high proportion of 3,882 transcripts compared to other conditions (Figure 5A). This likely reflects the critical transitional and reactivation of biological processes as the plant holobiont progresses toward full rehydration. In contrast, the D and FR samples shared only 195 transcripts, suggesting a reduction in transcriptional activity during desiccation stress. A total of 2,986 DATs were identified between two conditions, with 1,041 upregulated under drought stress and 1945 upregulated during rehydration (Figure 5B). The higher number of DATs in rehydrated root samples compared to dehydrated ones indicates a resumption of microbial biochemical activities upon water availability. This highlights the dynamic transcriptional responses of the root-associated microbiome during the transition from desiccation to rehydration.

### 3.5 Role of carbohydrate metabolism in desiccation

A significant proportion of DATs elevated under drought stress were also related to carbohydrate metabolism. These DATs included genes encoding glycogen synthase, 6-phosphogluconate dehydrogenase, gluconate transporter, stress response protein ysnF, glucosyl-3-phosphoglycerate synthase, sucrose-phosphate synthase, ABC transporters, malto-oligosyltrehalose synthase, trehalose-6-phosphate synthase, and trehalose synthase (Figure 5C; Supplementary Figure S2; Supplementary Table S2). Desiccation induced the expression of genes involved in glycogen and trehalose biosynthesis. The latter consists of three distinct bacterial enzymes, namely malto-oligosyltrehalose synthase, trehalose synthase, and trehalose-6-phosphate synthase, whereas only glycogen synthase was detected for glycogen biosynthesis. Glycogen serves as both a carbon reservoir and a regulator of cell size under drought stress (Sekar et al., 2020) and is expressed in Pseudomonadota spp. during desiccation (Cytryn et al., 2007). Similarly, trehalose accumulation is characteristic of several of desiccation-tolerant organisms and has been reported in microorganisms, tardigrades, and resurrection plants such as *M. flabellifolia* and *Selaginella lepidophylla* (Asami et al., 2019; Avonce et al., 2006; Dace et al., 2023; Drennan et al., 1993; Farrant et al., 2009; Hernández-Fernández et al., 2022; Moore et al., 2007; Nguyen et al., 2022; Reina-Bueno et al., 2012; Yobi et al., 2013). The upregulation of trehalose biosynthesis genes (*TPS, TS,* and *TreY*) in bacteria suggests a case of convergent evolution, where similar desiccation tolerance mechanisms have emerged across diverse phylogenetic lineages (Figure 6).

Glycogen maintains cell size and acts as carbon storage in bacterial cells under drought stress (Sekar et al., 2020) and is expressed in *Psedomonadota* spp. under drought stress (Cytryn et al., 2007). The accumulation of trehalose is characteristic of several desiccation tolerant organisms and was previously detected in tardigrades, microorganisms and resurrection plants such as *M. flabellifolia* and *Selaginella lepidophylla* (Avonce et al., 2006; Drennan et al., 1993; Farrant et al., 2009; Hernández-Fernández et al., 2022; Moore et al., 2007; Nguyen et al., 2022; Reina-Bueno et al., 2012; Yobi et al., 2013). In contrast, genes that encode trehalose hydrolysis proteins and sucrose synthase were upregulated under rehydrated conditions, indicating that trehalose was no longer required, and sucrose might serve as a carbon source. Desiccation induced increases in transcripts involved in the energy generation pathways including pentose phosphate, glycolysis, glyoxylate shunt and oxidative phosphorylation pathway. Accumulation of these transcripts might be a preparation of cells for repair during rehydration. However, the upregulation of pentose phosphate metabolism under dry conditions has also been reported in other bacterial species and is thought to protect the cell against oxidative stress (Cytryn et al., 2007; Srikumar et al., 2019). Our findings highlighted changes in the expression of key drought-responsive genes, likely critical for responding to desiccation tolerance in the microbiome and possibly the host plant as well. Microbial defense mechanisms could influence the host plant, contributing its responses to drought (Rais et al., 2017).

### 3.6 Transcriptional response upon rehydration

The most abundant DATs under rehydration conditions were associated with protein metabolism, accounting for 23.75% of the total DATs (Supplementary Figure S2). Notably, rehydration led to a significant increase in drought-responsive genes such as catalase-peroxidase, FMN-binding glutamate synthase family protein, HSP70, and glutamate synthase in the rehydrated samples (Figures 5C,D). Genes involved in the regulation of salicylic and jasmonic acid pathways, such as salicylaldehyde dehydrogenase and PAP2 family proteins were enriched, likely reflecting. The transition phase from PR (43 ± 7.72% RWC) to desiccation stress. Our results are consistent with previous studies, showing upregulation of drought-response pathways such as glutathione metabolism during rehydration conditions in resurrection plants (Liu et al., 2018; Ma et al., 2015). Additionally, genes associated with carbohydrate metabolism including trehalose utilization, glutamine-fructose-6-phosphate transaminase activity, and carbohydrate transporter proteins (Figure 5D; Supplementary Figure S2), with the upregulation of trehalose utilization genes indicates that it is no longer required in favorable conditions, consistent with previous findings (Reina-Bueno et al., 2012).

The rehydrated samples also exhibited an enrichment of DATs linked to the biosynthesis of the outer membrane, including pathways such as flagellum assembly, peptidoglycan synthesis, desaturase, long-chain fatty acid acyl-CoA synthetase and bacterial chemotaxis (Figure 5D). These transcripts, alongside the upregulation of murein hydrolase enzymes, are crucial for peptidoglycan sacculus growth and bacterial viability (Typas et al., 2012; Walter and Mayer, 2019). Furthermore, the COG analysis of rehydrated samples indicated that DATs were predominantly affiliated with Acidobacteria and Pseudomonadota, which were significantly abundant in rehydrated roots (Supplementary Table S2). These findings suggest that rehydration modulates the recolonization of drought-sensitive bacteria through enhanced polysaccharide biosynthesis and chemotaxis to the root. Rewatering likely stimulated repair of desiccation induced damaged and *de novo* peptidoglycan biosynthesis in bacterial cells within and around the roots of *M. flabellifolia*.

Genes involved in plant-microbe interactions, such as those related to chemotaxis, fatty acid hydrolase and flagellar motility, were upregulated during rehydration, possibly facilitating bacterial colonization on the root surface (Compant et al., 2021) and movement within the viscous rhizosphere as roots absorbed water (Figure 6). The activation of the exopolysaccharide biosynthesis pathway may further enhance bacterial motility, providing a competitive advantage for root colonization (Scharf et al., 2016). Additionally, bacteria may improve their interactions with *M. flabellifolia* by expressing extracytoplasmic function (ECF) sigma factor genes, which enhance symbiotic efficiency (Sheibani-Tezerji et al., 2015). Most of enriched transcripts after rehydration were linked to repair and rebuilding damaged membranes, replenishing energy, and carbon stores.

In prokaryotic cells, polyamines such as spermidine and putrescine play a critical role in DNA packaging during cell cycling (Souzu, 1986). Interestingly, the upregulation of these genes during rehydration was observed (Figure 6). Polyamines are also known to stabilize bacterial spheroplasts and protoplasts against osmotic stress (Hernández-Fernández et al., 2022). Our findings revealed the upregulation of DATs associated with nutrient acquisition, including phosphatases and nitrate reductases, which are pivotal for phosphorus mobilization and nitrogen metabolism, respectively (Figure 6). These results suggest that root-associated bacteria may enhance nutrient acquisition in *M. flabellifolia* under fully hydrated conditions by improving phosphorus availability and facilitating nitrogen assimilation, aligning with previous observations in other plant species (Berger et al., 2020; Cabugao et al., 2017; Tebele et al., 2024).

## 4 Conclusion

In conclusion, the intricate interaction between plants and root-associated bacteria is an enigmatic phenomenon that requires further investigation. These bacterial communities could offer a promising strategy to enhance plant tolerance to various abiotic stresses, particularly through microbial inoculants and biostimulants in agricultural systems. This study used metatranscriptomics to characterize the bacterial communities associated with *M. flabellifolia* roots and examine their transcriptional responses under desiccation stress. Desiccation led to the upregulation of numerous stress-responsive genes, including those involved in cell signaling, molecular chaperone activity, kinases, antioxidant defense, trehalose synthesis, and transportation. These findings suggest that *M. flabellifolia* recruits microbes that are also desiccation-tolerant, indicating potential convergent evolution between the plant and its microbiome. Future research should consider culture-dependent methods to identify key bacterial species and apply these isolates to improve drought resistance in more vulnerable crops. Altogether, this study highlights the functional roles of root-associated bacteria at the transcriptional level and their potential to improve plant resilience, with implications for agriculture.

## Data Availability

The datasets presented in this study can be found in online repositories. The names of the repository/repositories and accession number(s) can be found in the article/Supplementary material.

## Footnotes

^1^ https://jgi.doe.gov/data-and-tools/software-tools/bbtools

^2^ https://multiqc.info

^3^ https://www.biobam.com/omicsbox
