# Supplementary material for: Microbial survival strategies in desiccated roots of Myrothamnus flabellifolia
Source: Front Microbiol. 2025 Mar 28;16:1560114. doi: 10.3389/fmicb.2025.1560114 (PMC11985526; doi:10.3389/fmicb.2025.1560114)
Supplement: Supplementary file 1 [file Table_1.docx]

**Microbial survival strategies in desiccated roots of *Myrothamnus flabellifolia***

Shandry M. Tebele^1^, Rose A. Marks^1,2^, and Jill M. Farrant^1*^

^1^Department of Molecular and Cell Biology, University of Cape Town, Rondebosch 7701, South Africa

^2^ Department of Plant Biology, University of Illinois Urbana-Champaign, Urbana, Illinois, 61801, USA

Supplementary Material

Table S1. A summary of statistical analyses of root metatranscriptomic data.

| **Species** | | | | | | | | |
| --- | --- | --- | --- | --- | --- | --- | --- | --- |
| **Organism Name** | **baseMean** | **controlMean** | **experimentalMean** | **log2FoldChange** | **lfcSE** | **stat** | **pvalue** | **padj** |
| *Acinetobacter baumannii* | 81874.58 | 100166.610 | 63582.56 | -0.6557143 | 0.1936222 | -3.386566 | 0.0007077 | 0.0055928 |
| *Ktedonobacter racemifer* | 48649.80 | 37937.307 | 59362.30 | 0.6459169 | 0.3637125 | 1.775900 | 0.0757495 | 0.1967912 |
| *Streptomyces sp.* | 6608.4330 | 6595.1540 | 6621.7119 | 0.005932899 | 0.1544707 | 0.03840792 | 9.693624E-01 | 9.830555E-01 |
| *Bradyrhizobium sp.* | 4925.8932 | 3682.2544 | 6169.5321 | 0.744511194 | 0.2545827 | 2.92443742 | 3.450795E-03 | 1.983425E-02 |
| *Thermogemmatispora carboxidivorans* | 18122.42 | 13324.619 | 22920.21 | 0.7824749 | 0.3650272 | 2.143607 | 0.0320644 | 0.1065079 |
| *Leptolyngbya sp.* | 2830.6910 | 3360.6565 | 2300.7256 | -0.547243933 | 0.1691053 | -3.23611274 | 1.211695E-03 | 8.537963E-03 |
| Acidobacteriaceae bacterium | 13187.06 | 6962.772 | 19411.35 | 1.4790009 | 0.2391456 | 6.184520 | 0.0000000 | 0.0000000 |
| *Rhizobium sp.* | 1865.3539 | 1811.6315 | 1919.0764 | 0.083953225 | 0.1638889 | 0.51225698 | 6.084712E-01 | 7.607139E-01 |
| *Sphingomonas sp.* | 1960.0749 | 1977.0280 | 1943.1218 | -0.023028272 | 0.1153519 | -0.19963491 | 8.417661E-01 | 9.163687E-01 |
| *Paenibacillus sp.* | 13171.6376 | 16628.7086 | 9714.5666 | -0.775436304 | 0.2338759 | -3.31558821 | 9.145047E-04 | 6.782782E-03 |
| **Gene** | | | | | | | | |
| NADH dehydrogenase subunit D | 8259.7274 | 13030.7430 | 3488.7118 | -1.901170288 | 0.28798519 | -6.601625228 | 4.066745E-11 | 2.091704E-09 |
| ATP synthase subunit alpha | 14094.313 | 21627.06 | 6561.568 | -1.7207460 | 0.2730020 | -6.303053 | 0.0000000 | 0.0000000 |
| hypothetical protein, partial | 13589.964 | 17863.86 | 9316.071 | -0.9392467 | 0.2493905 | -3.766168 | 0.0001658 | 0.0013827 |
| molecular chaperone DnaK | 12705.677 | 18053.46 | 7357.894 | -1.2948440 | 0.3555184 | -3.642129 | 0.0002704 | 0.0020627 |
| cytochrome c oxidase subunit I | 11498.263 | 17972.12 | 5024.410 | -1.8387912 | 0.3066379 | -5.996620 | 0.0000000 | 0.0000001 |
| molecular chaperone HtpG | 4409.0178 | 6563.5007 | 2254.5350 | -1.541499609 | 0.37084332 | -4.156740910 | 3.228197E-05 | 3.497443E-04 |
| molecular chaperone GroEL | 4302.1224 | 2749.7590 | 5854.4859 | 1.090524255 | 0.34340663 | 3.175606268 | 1.495237E-03 | 8.417633E-03 |
| ABC transporter ATP-binding protein | 3572.6622 | 2842.9158 | 4302.4085 | 0.597352517 | 0.12764494 | 4.679797715 | 2.871581E-06 | 4.414732E-05 |
| glutamate synthase large subunit | 2070.0429 | 2290.4592 | 1849.6266 | -0.308197927 | 0.19921728 | -1.547044176 | 1.218526E-01 | 2.424198E-01 |
| peptidylprolyl isomerase | 1248.3097 | 1456.4222 | 1040.1971 | -0.484903910 | 0.25021532 | -1.937946519 | 5.262975E-02 | 1.334837E-01 |
| **Pathway** | | | | | | | | |
| Respiration | 92004.26 | 129284.89 | 54723.62 | -1.2403255 | 0.2402401 | -5.1628582 | 0.0000002 | 0.0000009 |
| Protein Metabolism | 87898.68 | 84918.49 | 90878.87 | 0.0979247 | 0.0895018 | 1.0941081 | 0.2739076 | 0.3361593 |
| Carbohydrates | 59710.87 | 60398.90 | 59022.85 | -0.0332742 | 0.1252074 | -0.2657529 | 0.7904295 | 0.7904295 |
| RNA Metabolism | 37519.34 | 39718.22 | 35320.46 | -0.1692456 | 0.1008565 | -1.6780832 | 0.0933309 | 0.1326281 |
| Amino Acids and Derivatives | 27723.78 | 25978.56 | 29469.00 | 0.1818033 | 0.0538674 | 3.3750166 | 0.0007381 | 0.0013286 |

**
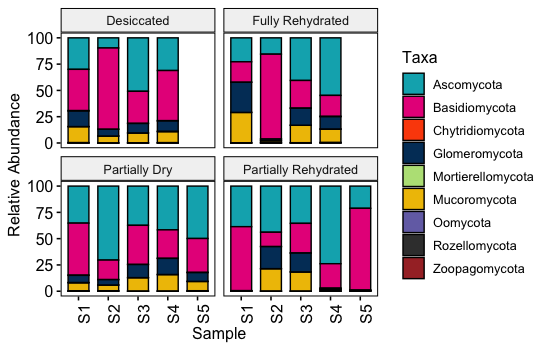
**

**Figure S1**. Relative abundance of fungal phyla associated with metatranscriptome root transcripts across four conditions.


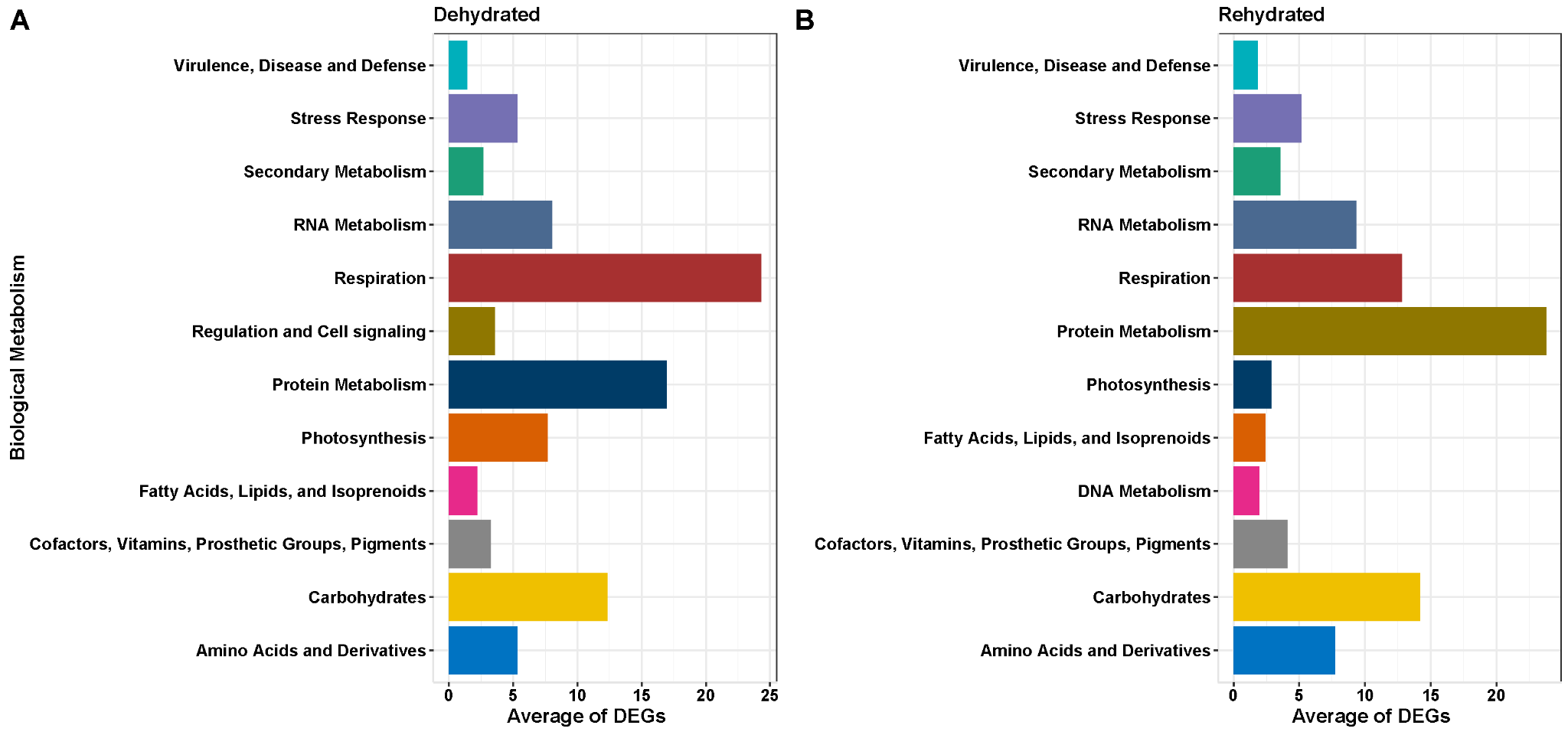


**Figure S2**. Functional assignment of bacterial transcripts. A) The proportion of DEGs under drought stress and B) rehydration conditions.

**Table S2**. COG function and associated taxonomic of the top 50 most significant transcripts with a P < 0.001 under dehydrated and rehydrated conditions.

| **Gene ID** | **COG Gene** | **baseMean** | **log2FoldChange** | **p-adj** | **Taxa** |
| --- | --- | --- | --- | --- | --- |
| **Dehydrated** | | | | | |
| k91_298508_1 | CsbD family protein | 516.532031 | -4.0636987 | 1.04E-50 | Methylobacteriaceae |
| k91_409495_1 | CBS domain-containing protein | 403.869509 | -2.9912475 | 5.95E-49 | Asterids |
| k91_983256_1 | cyclase | 662.937402 | -2.6499532 | 3.57E-46 | Actinobacteria |
| k91_1281742_1 | thiamine pyrophosphate-requiring protein | 1262.37832 | -4.1767779 | 1.84E-41 | Chloroflexi |
| k91_845164_1 | type 1 glutamine amidotransferase | 788.186432 | -3.3741407 | 6.32E-37 | Actinobacteria |
| k91_1452504_2 | DNA starvation/stationary phase protection protein | 463.339247 | -3.8278342 | 1.26E-33 | Chloroflexi |
| k91_1760887_1 | alpha-1,4-glucan--maltose-1-phosphate maltosyltransferase | 306.696036 | -3.8228572 | 4.95E-33 | Actinobacteria |
| k91_374216_1 | isoaspartyl peptidase/L-asparaginase | 421.910459 | -3.8003021 | 4.95E-33 | Actinobacteria |
| k91_878415_1 | SigB/SigF/SigG family RNA polymerase sigma factor | 159.703181 | -2.2687944 | 1.72E-31 | Acidobacteria |
| k91_867851_1 | NADP-dependent succinic semialdehyde dehydrogenase | 344.51143 | -5.1612928 | 2.42E-31 | Agaricales |
| k91_1338059_1 | DUF1236 domain-containing protein | 194.055745 | -4.5357471 | 4.25E-31 | Alphaproteobacteria |
| k91_687873_1 | DUF2795 domain-containing protein | 163.782081 | -4.6253997 | 1.11E-29 | Actinobacteria |
| k91_250190_1 | ornithine decarboxylase | 120.413557 | -4.0593249 | 1.12E-28 | Agaricales |
| k91_1941786_1 | DUF1360 domain-containing protein | 41.2765681 | -4.6967151 | 1.59E-28 | Actinobacteria |
| k91_615193_1 | glycogen-branching enzyme | 170.181858 | -2.4183341 | 1.05E-27 | Chloroflexi/Cyanobacteria/Glomerellales/Actinopolysporales/Desulfovibrionales |
| N/A | DUF3416 domain-containing protein | 310.735835 | -3.8042511 | 1.13E-26 | N/A |
| k91_1882594_1 | catalase HPII | 916.947055 | -3.283786 | 1.35E-26 | Chloroflexi |
| k91_191260_1 | YihY/virulence factor BrkB family protein | 169.388878 | -2.8206678 | 1.64E-26 | Acidimicrobiia |
| k91_555370_1 | hemerythrin | 110.694383 | -2.8191745 | 4.10E-26 | Actinobacteria/Cyanobacteria/Cyanothece |
| k91_958548_1 | protein-L-isoaspartate O-methyltransferase | 110.657471 | -1.8869428 | 2.50E-25 | Dothideomycetes/asterids/Brassicales |
| k91_391950_1 | photosystem reaction center subunit H | 326.150282 | -3.1027519 | 2.29E-24 | Micromonosporales |
| k91_1205183_1 | DUF2382 domain-containing protein | 161.222458 | -3.1770835 | 4.85E-23 | Acidobacteria |
| k91_298508_1 | MULTISPECIES: CsbD family protein | 54.4490408 | -3.5501619 | 1.07E-22 | Methylobacteriaceae |
| k91_1837332_2 | phosphosulfolactate synthase | 64.1821517 | -2.1311331 | 1.49E-22 | Bacteria |
| k91_512729_1 | transpeptidase | 137.850686 | -5.1520841 | 2.45E-22 | Chloroflexi/Pseudonocardiales/Acidimicrobiia |
| k91_610115_1 | CHAD domain-containing protein | 138.845089 | -3.3454495 | 3.82E-22 | Actinobacteria/Intrasporangiaceae |
| k91_1062562_1 | ferritin-like domain-containing protein | 484.306755 | -2.9645767 | 4.70E-22 | Acidobacteriia/Xanthomonadales |
| k91_1506508_1 | transaldolase | 1728.15776 | -1.6249579 | 2.17E-17 | Acidobacteriia |
| k91_1111686_1 | DUF1206 domain-containing protein | 72.4035004 | -3.5674683 | 5.07E-17 | Actinobacteria |
| k91_374216_1 | asparaginase | 66.3552925 | -2.5884297 | 5.30E-17 | Actinobacteria/Asterids |
| k91_947556_1 | YtxH domain-containing protein | 290.265078 | -3.2799797 | 3.00E-21 | Acidobacteria |
| k91_868149_2 | 1,4-alpha-glucan branching enzyme | 224.788343 | -2.0889192 | 3.71E-21 | Planctomycetes/Actinobacteria/Acidobacteriia/ |
| k91_518415_1 | DUF1942 domain-containing protein | 115.91679 | -4.7278614 | 8.86E-21 | Mycobacteriaceae |
| N/A | BON domain-containing protein | 180.340521 | -1.894079 | 9.59E-21 | N/A |
| k91_220636_1 | ATP-dependent DNA ligase | 279.271271 | -2.4871444 | 1.79E-20 | Pseudonocardiales |
| k91_1795709_1 | DUF4142 domain-containing protein | 228.84645 | -3.1887666 | 4.06E-20 | Acidobacteria |
| k91_752096_1 | LLM class F420-dependent oxidoreductase | 1085.49075 | -1.8786248 | 7.54E-20 | Chloroflexi |
| k91_845164_1 | MULTISPECIES: type 1 glutamine amidotransferase | 56.9532004 | -4.0747855 | 9.04E-20 | Actinobacteria/Chloroflexi |
| k91_688082_1 | thiamine pyrophosphate-requiring protein, partial | 30.0854112 | -5.5774664 | 2.44E-19 | unclassified Bacteria/Actinopolysporales |
| k91_1771606_1 | protease | 397.633166 | -1.7896437 | 5.63E-19 | asterids |
| k91_1253011_1 | DUF892 domain-containing protein | 210.730329 | -2.3246033 | 8.27E-19 | Acidobacteria |
| k91_987651_1 | beta-phosphoglucomutase family hydrolase | 58.6451185 | -2.0978261 | 1.68E-18 | Actinobacteria |
| k91_836742_1 | gas vesicle protein | 113.095422 | -2.7373956 | 3.98E-18 | Bacillus/Nocardiaceae |
| **Rehydrated** | | | | | |
| k91_182886_1 | glutamine synthetase type III | 41.0427239 | 3.23330875 | 1.64E-21 | Planctomycetes |
| k91_199743_1 | outer membrane protein assembly factor BamA | 60.0840792 | 3.75691164 | 2.69E-21 | Enterobacter |
| k91_438159_1 | Flp family type IVb pilin | 40.5864946 | 3.97840492 | 1.75E-20 | Bradyrhizobiaceae |
| k91_1454583_1 | EvpB family type VI secretion protein | 48.0552393 | 4.08852905 | 6.96E-20 | Acidobacteria |
| k91_1943812_1 | flagellin | 118.641931 | 3.55224035 | 4.21E-18 | Acidimicrobiia/Acidobacteria |
| k91_1235964_1 | TonB-dependent receptor | 366.851888 | 2.82756323 | 4.57E-18 | Acidobacteria/Chlorobi |

**Data availability**

The generated datasets for this study can be found in the European Nucleotide Archive (ENA)

database under the accession number PRJEB83242.
